# “The meal is an activity involving at least two people”—Experiences of meals by older persons in need of elderly care

**DOI:** 10.1002/nop2.387

**Published:** 2019-10-29

**Authors:** Sigrid Odencrants, Karin Blomberg, Anne‐Marie Wallin

**Affiliations:** ^1^ Department of Nursing Faculty of Medicine and Health School of Health Sciences Örebro University Örebro Sweden

**Keywords:** experiences, interviews, meal, meal situation, older persons, qualitative study

## Abstract

**Aim:**

A shift of focus on older persons’ nutrition has occurred, from focusing on nutrition status to focusing on the whole meal. There is lack of studies on how older persons experience meals. Knowledge is needed to enhance a meal with dignity and pleasure, but also to prevent deterioration in nutrition. The aim was to describe meals from the perspective of older persons in need of elderly care.

**Design:**

A descriptive qualitative study.

**Method:**

Semi‐structured interviews (N = 18) were conducted and analysed using thematic analyses.

**Result:**

Three themes were identified: *The meal is an activity which involves at least two persons*, *The meal relates to habits and traditions* and *The meal seldom gives possibilities to make individual choices*. It was obvious that older persons who live in nursing homes miss the opportunity to decide what to eat, when to eat and with whom to eat. Attention must be paid to listening to older persons to enhance mealtime with dignity and autonomy.

## 1 INTRODUCTION

Several studies have described the importance of optimal nutrition for older persons’ health and quality of life (Milte & McNaughton, 2016). Thus, it is necessary to increase knowledge and understanding of what affects and influences older persons’ nutrition. Factors which lead to poorer nutritional status among older persons are described in literature, such as intrinsic and extrinsic determinants, including biological, societal and environmental factors (Morais et al., 2013). Biological factors include, for example impaired salivation, dry mouth, chewing and swallowing difficulties, impaired vision and changes in taste and smell that can have a negative effect on appetite and eating (Holst, Rasmussen, & Laursen, 2012). Psychological factors include, for example, depression, other mental and cognitive problems (Faxén‐Irving, Basun, & Cederholm, 2005) and societal factors that might influence nutritional status are, for example eating alone (Martin, Kayse‐Jones, Stotts, Porter, & Sivarajan, 2005; Wright, Hickson, & Frost, 2006), isolation (Hughes, Bennett, & Hetherington, 2004) and economic factors (Donini et al., 2013). Other risk factors reported are the use of meals on wheels to replace cooking independently, long overnight fast factors and few eating episodes (Söderström et al., 2013). It is also reported that the distribution of food and meals in the care of older persons is often tailored to the surrounding practical organization factors rather than to older person's needs and wishes (Mattsson‐Sydner & Fjellström, 2006) and this might affect the older person's nutrition.

Previously, the aim for public meals, such as meals for older persons, has been to nourish and give energy (Persson Osowski, 2012). This is in contrast to restaurants where eating is related to pleasure (Lupton, 1998). This difference can also be seen in relation to the focus for nutritional interventions for older persons which has shifted from nutritional content and diet to be more focused on the whole meal (Magnusson Sporre, Jonsson, & Pipping, 2016; Reimer & Keller, 2009). For example, interventions aimed to maintaining optimal nutritional status are primarily based on “ordinary food” which can be supplemented with energy and protein enriched food. Other interventions that are described include feeding assistance (Heaven, Bamford, May, & Moynihan, 2013) and creation of a trustful atmosphere (Mamhidir, Karlsson, Norberg, & Kihlgren, 2007). However, Murray (2006) suggests that interventions do not always need to be “high tech”; *prioritising meals* is one of the simplest and most effective solutions.

Mealtimes are described as a major social event and an activity for older persons to interact with others (Curle & Keller, 2010). To eat was described not only as an activity for refilling energy, it was also described as a part of the “meal occasion” that provided comfort, especially for institutionalized older persons (Edfors & Westergren, 2012). A study, which analysed how staff and residents shaped mealtimes according to frames and social scripts, reported a dominant institutional frame, difficulties in introducing private frames and problems with creating home‐like meal situations (Harnett & Jönsson, 2017). According to the literature, the importance of enhancing mealtime experiences by listening to older people and incorporating strategies to increase the food intake for elderly people has been reported (Mahadevan, Hartwell, Feldman, Ruzsilla, & Raines, 2014). Collaboration, knowledge and positive attitudes towards nutrition among the caregivers are important, as are empathy, trust and confidence between individual older persons and caregivers (Mahadevan et al., 2014).

The concept of the meal is difficult and complex, and it cannot be studied from only one perspective (Sobal & Bisogini, 2009). The concept meal situation has therefore been developed to capture more than a single event for a meal. Since the proportion of older persons in Europe continues to increase (Gaag & Erf, 2008), meals in public care are important issues for countries with a growing elderly population (Magnusson Sporre et al., 2016). Data describing older persons’ experiences of meals are therefore important for gaining knowledge to facilitate useful meal interventions, irrespective of living conditions. Therefore, the aim of this study was to describe experiences of meals from the perspective of older persons in need of elderly care.

## 2 METHODS

The study has a qualitative approach with a descriptive design (Polit & Beck, 2017), based on semi‐structured interviews with older persons having different needs of support by elderly care. The study is a part of a larger project titled Prio‐Food (Priority Food), with the overall aim of identifying, assessing and supporting nutrition among older persons.

### 2.1 Sample and data collection

Participants were as selected among older persons in a Swedish community with 11,000 inhabitants, of whom 2,944 individuals were ≥65 years old. Inclusion criteria were older persons (≥65 years of age) in need of elderly care, who participated in a previous quantitative data collection designed to map nutritional status for older persons in the community. Altogether 210 older persons were invited and 64 agreed to participate. All of them were asked by the researchers if they were interested in participating in a subsequent individual interview and 25 older persons expressed an interest in doing so.

Finally, a convenience sample of 18 older persons, 11 women and 7 men with a median age of 84 years (range 70–94), were included in the study. Reasons for not participating were lack of interest, lack of time or energy, impaired health or death. Of the 18 persons included, five were identified as being at risk for malnutrition. Using the instrument Mini Nutritional Assessment (MNA) (Rubenstein, Harker, Salvà, Guigoz, & Vellas, 2001), six were identified as malnourished (four men and two women), of whom four lived on their own and two in community living. The concept "community living” is hereafter used for older persons living in nursing homes or senior housing. Participants’ characteristics are presented in Table 1 and participants’ nutritional status are shown in Table 2.

**Table 1:** Participants’ characteristics (*N* = 18)

Demographic variables	*N* = 18	Median, (range)
Age (years)		
Female (*N* = 11)		84 (74–94)
Men (*N* = 7)		84 (70–90)
Marital status		
Single	3	
Married	5	
Widowed	10	
Living		
Own house/apartment	10	
Senior housing^a^	4	
Nursing home	4	
Food administration		
Nursing home	4	
Meals on wheels	4	
Homemade	10	

a Senior housing, own apartments with daytime staffing and an age limit of >55 years.

**Table 2:** Anthropometric parameters for participants (*N* = 18)

Weight, BMI, nutritional status	
Weight (kg)	
Total sample (*N* = 18)	71 (47–92)
Female (*N* = 11)	67 (47–81)
Men (*N* = 7)	84 (54–92)
Body mass index	
Total sample (*N* = 18)	27 (16–40)
Female (*N* = 11)	26 (16–36)
Men (*N* = 7)	27 (16–40)
Nutritional status (MNA)	
Total sample (*N* = 18)	23 (13–26)
Female (*N* = 11)	23 (13 –26)
Men (*N* = 7)	19 (13–26)

Note

Abbreviation: MNA, Mini Nutritional Assessment.

<17 points = malnourished, 17–23.5 points = at risk for malnutrition, ≥24 points = well nourished.

### 2.2 Individual interviews

An interview guide with the aim of eliciting the participants’ experiences of meals was developed. Individual semi‐structured interviews were conducted by two of the authors (SO & AMW), who were very experienced in interviewing older persons, at a place and time chosen by the participants. The interviews were divided between the interviewers and there were no relationships between the older persons and the interviewers. All the interviews were conducted in the older persons’ homes and lasted between 25–60 min. The interviews started with an open question: “How has it been with the meal today?” To reach a deeper understanding, probing questions were asked, such as “Could you describe this in more detail?” The interviews were conducted as a conversation, and the researchers adjusted and explained the questions to the participants if anything was perceived as unclear (Patton, 2002). The interviews were all audio‐recorded with the participants’ permission and transcribed verbatim by a secretary experienced in transcription.

### 2.3 Ethical considerations

The project was approved by the Regional Ethical Board of Uppsala, Sweden (reg. no. 2011/311). The participants were carefully informed both verbally and written about the study and their participation was requested before the interviews. They were informed about their right to withdraw without personal consequences. Written consent was obtained, with family members signing for those participants who could not sign for themselves. Since nutrition can be a sensitive and private subject to talk about, there was an opportunity for participants to contact the researchers if they desired. All data were coded, and personally identifiable information was removed. The principles of the Declaration of Helsinki were followed through all phases in the research process (World Medical Association (WMA), 2013).

### 2.4 Analysis

Data were analysed by all authors using the principles of thematic analysis according to Braun and Clarkes` method (Braun & Clarke, 2006). We determined to provide a rich description of the whole data set, which can be helpful when a topic is under‐researched, without being dependent on a specific theory. This study adopted an essentialist/realist approach for investigating how the participants experienced, understood and expressed their thoughts about the meal. An inductive approach was chosen and themes were identified at a semantic level, that is, the surface meanings expressed by participants. The analysis followed the six phases described. After a first individual reading of 10 of the interviews, notes and ideas for themes were made in the margins of text. The read‐through process generated initial codes that formed the basis for analysis of the eight remaining interviews. The codes represented content from the data relevant to the aim of the study and the coding process consisted of organizing or structuring the data into groups. Repeated patterns in the data set and codes then became the basis for the themes. When all data were coded, the sorting of codes with similar content into subthemes and main themes began. During this phase, some codes did not seem to fit anywhere and had to be discussed. Reviewing and refining the data were a prerequisite to ensure that the data fitted within the themes and themes were discrete. This phase gave an indication of what the themes were and of the overall story told by the data. Finally, we defined and named the themes to clarify them. The analysis moved back and forth between the themes and subthemes until the full meaning of the themes was revealed (Creswell, 2000; Shenton, 2004). Three themes were identified from the analysis and these are used below to structure the presentation of the results. Examples of the codes, subthemes and themes are presented in Table 3. Quotations from participants are used to illustrate the findings and presented in italics. Basic identification of the participants’ quotations is given with [NH] for Nursing home, [SH] for Senior housing and [OH] for Own home. A bilingual Swedish–English speaker translated the quotes from Swedish to English.

**Table 3:** Examples of analysis from codes to themes based on interviews with older persons

Codes	Subthemes	Themes
Get to get out of the room	Activity	
To get a little food and talk with the staff		
Getting food ready both activity and a pause		
It means a lot to have prepared one's own food from scratch	Prepare food	The meal is an activity and involves at least two people
Able to see ingredients and choose for myself		
If I’m being really honest I’d like to avoid preparing food		
Company—together	Company and pleasure	
That it is pleasant around me and I get to sit in peace and quiet		
High point—to be able to eat together		
To eat something, doesn't have to be special		
Ordinary food which I recognize	Recognition and memories	
Memories of mum, memories of mashed potatoes and carrots		
Farm life—eat at mid‐day—this is how I grew up and still do the same		
I’m surprised that they serve Christmas rice pudding all year round		
Prepared it differently—we old people have our own recipes	Homemade or ready‐made food	The meal relates to habits and traditions
Bought food doesn't taste of real food—tastes musty		
Never wasted anything, it's how I was brought up		
Eat food which is not scheduled for that day	Scheduled food	
Large portions		
Eat the same food two times a day		
Finish eating, but they start to tidy up when people start to go	The staff acts in the dining room	The meal is arranged according to the staff's preferences
Depends on who is working if the food is in serving dishes and you can help yourself		
Obviously, you'd like a little extra sometimes, but you can't have it		
One of the staff decides who sits where, but I can sit anywhere	Seating arrangement	
Scared of ending up in another group and not having the same friends		

## 3 FINDINGS

Three themes were identified from the analysis: “The meal is an activity and involves at least two persons,” “The meal relates to habits and traditions” and “The meal seldom gives possibilities to make individual choices.” The themes are described below.

### 3.1 The meal is an activity and involves at least two people

Preparing meals was described as an activity and a nice interruption to everyday life. Some participants described it as fun and enjoyable, as long as they had the energy for it. The opportunity of preparing food included aspects of being curious and trying out new recipes or styles of cooking. To still be able to prepare the meal was described as a pressure and some expressed a wish to be supported with ready‐cooked food some days. Preparing meals was for some related to the women's roles of being responsible for food shopping and preparing food for their families in the past. To have the opportunity to visit the grocery shop, to see and choose food for themselves and to be influenced by special offers or other things that influenced their shopping, were positive experiences.

When the participants were asked to express their thoughts about eating and meals, their answers varied from nothing special to very specific situations or types of food. Some described eating as necessary to avoid losing weight, to survive or to provide energy, or simply said that one must have something to eat. Participants described enjoying the food, of tasting something specific and described images of themselves eating: “Eating – you're hungry, you long for food” [SH]. Eating was not associated with anything in particular; it was an action everyone did every day and in the same way: “Yawn, chew and swallow” [OH]. Meals were described in terms of togetherness and being in company; having more than one person present was a prerequisite. Participants associated meals with a situation that was relaxed and leisurely. Eating together was a highlight of the day and an enjoyable experience. For one, meals were something that he looked forward to, partly because they meant that he could leave his room. Meals did not need to include remarkable food, but without tranquillity, the meal was worthless. One expressed, “That things are warm and comfortable around me. That I get to sit in peace and quiet” [NH].

### 3.2 The meal relates to habits and traditions

Sometimes, the food, or its name or smell, triggered strong memories, or inspired the imagining of a special food or dinner, thus creating expectations. When they ate it, however, they recognized neither its appearance nor the taste. The taste and appearance of the food were, as already mentioned, different from before, being expressed as “And so maybe you sit and long to get cabbage rolls today, then comes the box with some little white square and I had been counting on getting real cabbage rolls with cabbage leaves” [OH]. Participants had experience of shorter or longer periods of using meals on wheels; some had tried it earlier but had changed to another solution, such as ordering meals from restaurants or buying ready‐cooked food. The food was described as “not real and home‐cooked” [SH] in contrast to the food the participants had eaten previously, especially traditional food. These experiences were mostly associated with the taste of the food served, the perception that it was not freshly cooked and the inability to choose between different dishes as they wished. Nearly all, regardless of where they lived, specifically mentioned potatoes. They found it hard to understand how potatoes, which are “easy to cook” [NH & OH] could taste and look unrecognizable as potatoes. Small potatoes, which were often served, were associated with something “that used to be fed to pigs” [OH] (when the older person was young), but not with food that was served to humans. It was important to still be able to prepare food from scratch, since ready‐made food did not taste like the food they were used to.

Food served on anniversaries was described as “a lot of food – too much” [NH]; the participants were not used to having such large amounts of that kind of food. On those days, the food was tasty; however, and the staff were described as arranging the meals in the best way possible, even if the older people had their own personal wishes. One woman was upset by the frequent serving of a porridge made from rice, milk and butter and served with cinnamon, which is often served at Christmas in Sweden; she described this as “pushing the Christmas porridge, to serve it in season and out” [NH].

There were reflections by the participants living in community housing about the timing of the main meal or dinner. Participants mentioned finding it difficult to determine which of the meals was their main meal, since all the meals were so heavy. The habit of enjoying the main meal at noon, retained from their upbringing in an agricultural environment, was still important to some. The participants’ described the amount of food allocated, the order where they were served and wishes for a lighter meal in the evening, such as soup, fruit cream, sandwiches or omelettes. No matter what a meal tasted like, the food had to be eaten up; as many participants said, “We were brought up to eat what we were given; nothing got thrown away and it is like that now too”[OH].

### 3.3 The meal seldom gives possibilities to make individual choices

Overly large portions were often mentioned by those own home living who had experience of meals on wheels. They sometimes divided a portion between two meals, or shared it with someone else. The food was delivered in portions, three times a week, with each portion chilled and marked with the dates and days for eating. Some expressed uncertainty over whether they were “allowed” [OH] to alter the order where the meals were supposed to be eaten. By contrast, one participant said that he ate the food in whichever order he liked, regardless of whether this was “allowed.” Meals for participants in nursing homes were described as variously influenced, controlled or determined by the staff. Eating in the dining room was common. Some had the option of having breakfast in bed or in their rooms; some had a kind of buffet, which they referred to as a “hotel breakfast” [NH]. For many, the seating in the dining room was determined by staff. One mentioned insecurity over places, whom to sit next to and being worried about changes to the places in future without her being informed. The serving of food was a concern – “some have an unpleasant way of serving the food on the plate when they serve it” [NH] – and the staff did not care about the individual's preferred portion size. “That I get some smaller portions and then that I can take more again instead, then it tastes better. A big portion and you just sit there and try to get it down..... it tastes better if you don't get too big a portion” [NH]. Porridge cooked by tired night staff, or inexperienced or uninterested staff was described as a sad experience. Some reported not being allowed enough time to finish the meal, with staff beginning to clear away dishes and clean up, even though some of the older people had not finished eating. “It is better to get some peace and quiet when you eat and so on, that they don't take it away from you so quickly, so that you can sit and talk a little more with each other” [NH].

Participants expressed the wish to be able to influence or choose their food, perhaps choosing between salad and vegetables. The older persons were given few possibilities to affect the menu and had no opportunity to choose something different. One said: “School children and prisoners can do it; why can't we?”[NH]. One participant also described lack of participation in practical aspects such as “what to eat, when to eat and whom to eat with” [NH].

## 4 DISCUSSION

These findings illustrate that the meal is of great value for older persons from multiple perspectives. Meals are not just a source of energy. For our participants, a meal required at least two persons. Mealtimes give opportunities for social meeting and facilitate activity. This can be referred to the concept of meal as not only relating to the food and meal arrangements but also to socializing, the physical environment during the meal and individual needs and choices (Magnusson Sporre et al., 2016). The meals in our study were described as being together, a pause, recognitions and tastes and memories. The food itself was not so important for some of the participants, rather it was the environment and atmosphere in the dining room which created a personal feeling of pleasure and company. Magnusson Sporre et al (Magnusson Sporre et al., 2016) identified five main categories which were important for meals; *New values in the meal; Hospitality and service in the meal; Meal environment; Meal experience and Food quality in the meal*. These categories highlight the importance of including social dimensions such as respectful meetings between all involved in the mealtime and to see and meet the older persons’ needs and desires. It is also important to know how the room or food quality affects the meal experience and, finally, how our senses interpret and perceive the mealtime (Magnusson Sporre et al., 2016). All these categories could be linked to the meal situation for older persons in our study, since those categories can emphasize that a public meal has purposes other than just feeding. Both the categories “New values in the meal” and “Hospitality and service in the meal,” described by Magnusson Sporre et al. (2016), include the concept of a respectful meeting. In our study, however, participants described the opposite, perceiving disrespect in different contexts, especially during meals when staff started to clean up while they were still eating. Needs and choices of meal situations were influenced by staff and were experienced as a hindrance by older people. One expression was not having opportunities to influence “what to eat, when to eat and with whom to eat.” This indicated the importance of the older persons needing to be involved and to have an active role in their meals. Today, the concept of patient participation has been explored to highlight the role of the “patient” in health care (Angel & Frederiksen, 2015; Eldh, Ekman, & Ehnfors, 2010). Despite regulations by the law in Sweden (SFS 2014), older persons in Sweden were the least involved in their care compared with other countries (Vårdanalys, 2017). Thus, there is a need to further investigate how to achieve participation among older persons, especially in meal situations.

The experience of the meal and food was also found to be essential for both recognition and security. Traditional food and associated expectations might stimulate the appetite, for example when reading a menu, though some of our participants related that the food that was actually delivered did not fulfil their expectations in terms of either taste or appearance. Similar results are described where it is reported that food experienced in the past life affected the perception of food and meals in the present life (Edfors & Westergren, 2012). Our suggestion for health care might be to involve older persons in the composition of menus, or to give them more possibilities to choose between different dishes.

Another perspective on meal‐related situations (Odencrants, Ehnfors, & Grobe, 2005) includes activities related to meals, such as food shopping and preparation. Meal‐related situations were sparsely described in our data, although most of the participants lived in their own homes. Some had meals on wheels for distribution of meals and they did not actually shop and prepare food themselves. To still have opportunities to choose food in the grocery shop was perceived as being important, and this might be associated with better food intake. One solution to make it possible for older persons to visit the grocery shop could be to provide help with transport back and forth and support for buying food in the store. To see, smell, taste and be inspired in the shop was and might be for some, both an activity and also a reason to act as an independent person. Another solution could be to arrange for elderly people to meet up and prepare and enjoy a meal together. In one study, however, older persons who were still able to make and eat “homemade” food described preparing meals for themselves in positive terms relating to self‐image and roles. Different kinds of food and different ways of preparing it have strong connections to identity (Fischler, 1988). Our findings are similar to those from a study among older men, who talked about “cooking as a pleasure” (Kullberg, Björklund, Sidenvall, & Åberg, 2011). Women in our study mostly described preparing food in terms of pleasure and leisure, but also felt it could be a burden, possibly based on traditions and gender roles.

For the participants in the current study, meals were associated with an activity involving at least two persons. Eating alone was not a meal, and there may be a risk of insufficient intake of food. It was also important to have pleasant surroundings and peace and quiet. To be able to eat together was a high point in contrast to eating alone in front of the television. It is reported that people eat more when they eat together; having several people eating together influences the duration of the meal and people are tempted to eat more (Pliner & Bell, 2009). Magnusson Sporre et al (Magnusson Sporre et al., 2016) also state the importance of consensus and collaboration between staff and guests and also between staff and staff. A further study highlights the importance of educating staff in their responsibility to stimulate interactions between older persons and staff (Curle & Keller, 2010). It might be equally valuable to create similar conditions within groups of older persons. Even if older persons hardly interact with one another, staff might consider as a basic human need, the presumption that a meal involves more than one person. Therefore, it should be a fundamental aspect of care to ensure that not only the atmosphere should be calm and friendly, but also that there are possibilities for having company during meals (Blomberg, Wallin, & Odencrants, 2019).

The interviews with older persons in the nursing homes yielded more data compared with the interviews with older persons living in their own homes or senior housing, despite the fact that there were fewer older persons in our study living in nursing homes. To still be living and eating in their own homes or in senior housing can be viewed as more natural and eating in nursing homes then becomes “something arranged” or “something else.” Reasons for this might be that in the nursing home staff assist residents during mealtimes and routines are based on common needs, not on those of the individuals. Older persons are unfortunately expected to change their habits when they move into a nursing home and the differences can be described as a transition from one's own private habits to new collective routines in a “public meal” setting (Mattsson‐Sydner & Fjellström, 2006).

To be served food is often unavoidable in care situations, even though some older people in our study reported having the opportunity of serving themselves. However, the different ways that staff approached the meal were described as negatively influencing the meal. The older persons in the study were not able to choose where to sit, or to decide the size of portions or when to finish the meal. All these could be seen as being disrespectful behaviour by the staff. These behaviours detracted from the enjoyment of the meal, especially when the staff were pressuring the older persons to finish eating by removing dishes, wiping the tables and starting to wash the dishes. Staff attitudes, values and priorities concerning nutrition in the care of older persons are very important. A less enlightened value system among staff has been shown to be related to a more paternalistic view of care, while staff with a more developed value system prioritize autonomy, dialogue and the older person's preferences and dignity (Kjellström & Sjölander, 2014). Having staff who understand the socio‐cultural importance of the meal situation for older people may increase the older person's feeling of pleasure related to the meal, which can in turn promote eating (Philpin, Merrell, Warring, Gregory, & Hobby, 2011).

An ethnographic study in dining spaces for older persons and a survey among staff was recently conducted. According to the results, it was found that autonomy and personal control, comfort of a home‐like environment and opportunities for social interactions and relations were important for older people. The physical environment enabled personal support and fostered effective teamwork (Chaudhury, Hung, Rust, & Wu, 2017). A respectful, quiet and stress‐free environment should be a matter of course during the meal, no matter where the meal takes place, who is eating or what they are eating. Prioritizing a meal does not need to be driven by finances or large resources. Instead, it can be a new way of thinking and appreciating that new perspectives, knowledge or small changes in previous routines are valued and important. Understanding the value of a meal for older people may be the beginning of a new approach.

### 4.1 Strengths and limitations

Of the 25 older persons eligible for interviews, 18 agreed to participate, which can be considered sufficient for a qualitative study (Patton, 2002). A convenience sampling was used, which may have limited the representativeness of the older persons, due to a risk that those who might have other needs or perspectives disagreed to respond because of fragility, illness or impaired nutrition. The participants varied in terms of gender, age, marital status and living conditions. An interview guide was used, which gave the option for allowing the participants to speak freely, including discussing, reflecting and expressing their wishes. The interviews were conducted by two researchers and therefore the older persons may have been given different follow‐up questions. However, this can also give opportunities for capturing variations in experiences. Through the analysis process, we strove to be open to the text to try to avoid the interpretation being directed by any pre‐understanding of the subject area. Discussions between all the authors were held during all the phases of the analysis process. The aim was to ensure that there was agreement regarding the content until consensus was reached. This process may have strengthened the trustworthiness of the analysis process. Data saturation (Fusch & Ness, 2015) was reached when no additional new information could be obtained and when further coding was no longer practical and when there is enough information to enable replication of the study. Since no new data occurred in the last interviews, it was considered that data saturation was reached and no additional interview was needed.

## 5 CONCLUSION AND CLINICAL IMPLICATIONS

The meal is of great value for older persons from multiple perspectives, not only as a source of energy, but also an opportunity for social meeting and for facilitating activity. For our participants, a meal required at least two people. The meal was not affected so much by the food that was served; it was more important to feel confident and to have a calm atmosphere. The lack of opportunities for deciding what to eat, when to eat and with whom to eat might be an expected finding, since being able to still live at home allow for the opportunity of maintaining habits and decisions about meals. However, much more attention must be paid to listening to older persons’ experiences to avoid meals being based on staff preferences.

Findings from this study can be used for considering how to reprioritize care for older persons. Staff could focus more on individuals’ needs and understand that the social interactions, environment, habits and traditions should provide safety and comfort during meals. Our findings could be a basis for the development of care for older persons, since data gives voice to the older persons’ own experiences regarding meals. Staff play an important role in meal situations, especially for older persons living in nursing homes. The rights of older people to maintain their autonomy and to make their own choices should be a golden rule. Acknowledging this is of utmost importance, regardless of the carers’ professions, since they are responsible for older people in their care and meals and meal‐related situations are a fundamental aspect of the care process. Expectations and values of staff involved in mealtime care should be considered and it may be necessary to raise and encourage discussions and to introduce new routines. Care based on ethical principles whereby the older person is viewed as an autonomous person with personal preferences is important, even in the context of meals and meal‐related situations. The results of this study may have implications for the development of policy documents, regardless of the context of the care. The concept meal is complex and individual and for older, possibly vulnerable persons, there may be additional complicating factors which need to be addressed.

## CONFLICT OF INTEREST

None.

## AUTHORS’ CONTRIBUTIONS

SO, KB and A‐MW designed the study. SO and A‐MW collected the data. All authors analysed the data and prepared the manuscript.

## ETHICAL APPROVAL

The study was conducted in accordance with the Declaration or Helsinki. Approval was obtained from the ethics review board of Uppsala, Sweden (reg. no. 2011/311).
